# T Cell O‐GlcNAcylation Increases in Patients With Allergic Rhinitis After the Natural Pollen Season

**DOI:** 10.1111/cea.70311

**Published:** 2026-04-20

**Authors:** Eduardo I. Cardenas, Maryam Jafari, Emma Nilsson, Agnetha Karlsson, Marianne Petro, Monika Ezerskyte, Ola Winqvist, Lars Olaf Cardell

**Affiliations:** ^1^ Division of ENT Diseases, Department of Clinical Science, Intervention and Technology Karolinska Institutet Stockholm Sweden; ^2^ Department of Otorhinolaryngology Karolinska University Hospital Stockholm Sweden; ^3^ ABC Labs, Biomedicum Stockholm Sweden

## Abstract

- O‐GlcNAcylation is a potential biomarker of off‐season immune activation in allergic rhinitis.
- Seasonal changes in O‐GlcNAcylation mirror allergen‐driven T‐cell activation.

To the Editor,

1

Persistent inflammation outside the pollen season is a recognised but poorly defined feature of allergic rhinitis (AR). Allergic rhinitis is a common inflammatory disease of the nasal mucosa that affects a substantial proportion of the population and involves complex immune and inflammatory pathways. Recent studies highlight the mechanistic diversity of AR and the need for improved molecular characterisation of disease activity [1, 2].

However, the cellular and molecular mechanisms underlying this off‐season immune activation remain incompletely understood and objective biomarkers that capture persistent immune activity are lacking. Because T‐cell activation is closely linked to cellular metabolic reprogramming, immunometabolic pathways may provide informative indicators of ongoing allergic inflammation.

O‐GlcNAcylation (O‐GlcNAc), a nutrient‐responsive posttranslational modification controlling transcription, signalling and T‐cell activation, has not been examined in human allergic disease [3, 4]. Here, we identify T‐cell O‐GlcNA as a candidate dynamic marker that distinguishes AR patients from healthy individuals and reflects natural seasonal allergen exposure.

We studied two independent cohorts of birch‐ and/or timothy‐pollen‐allergic individuals recruited in Stockholm, Sweden. Cohort 1 included 13 AR patients and 12 healthy controls sampled in the fall after the natural pollen season. Cohort 2 included 10 AR patients and 10 healthy controls; AR patients were sampled during peak pollen exposure (May–June 2024) and again ≥ 3 months later, allowing within‐subject seasonal comparison. Exclusion criteria included recent infection, corticosteroid use, antihistamines, asthma, or chronic respiratory disease. Healthy controls tested negative for allergy.

Peripheral blood mononuclear cells were isolated by Ficoll separation. PBMC isolation was performed within 1 h after blood collection to minimise preprocessing variability. Standard multicolor flow‐cytometry procedures were applied, including live/dead discrimination, fixation/permeabilisation, Fc‐blocking and staining of surface markers and intracellular O‐GlcNAc. T‐cell subsets (TH1, TH2, TH17, TC1, TC2, TC17) were defined by CD3/CD4/CD8 expression and the transcription factors T‐bet, GATA3 and RORγt. An isotype control was used to determine O‐GlcNAc^+^ gating and calculate ΔMFI. Samples were acquired on an LSR Fortessa X20 and analysed using FlowJo 10.7.1. Statistical analyses were performed using paired or unpaired *t*‐tests (GraphPad Prism 10; *p* < 0.05).

PBMC profiling in Cohort 1 demonstrated that AR patients exhibited significantly higher O‐GlcNAc in CD4^+^ and CD8^+^ T cells compared with healthy controls, whereas B‐cell and NK‐cell O‐GlcNAc remained unchanged. Both O‐GlcNAc ΔMFI and the proportion of O‐GlcNAc^+^ cells were elevated in TH and TC populations (Figure 1A), indicating that T‐cell O‐GlcNAc is increased in AR even months after pollen exposure and may signify persistent off‐season immune activation.

**FIGURE 1:**
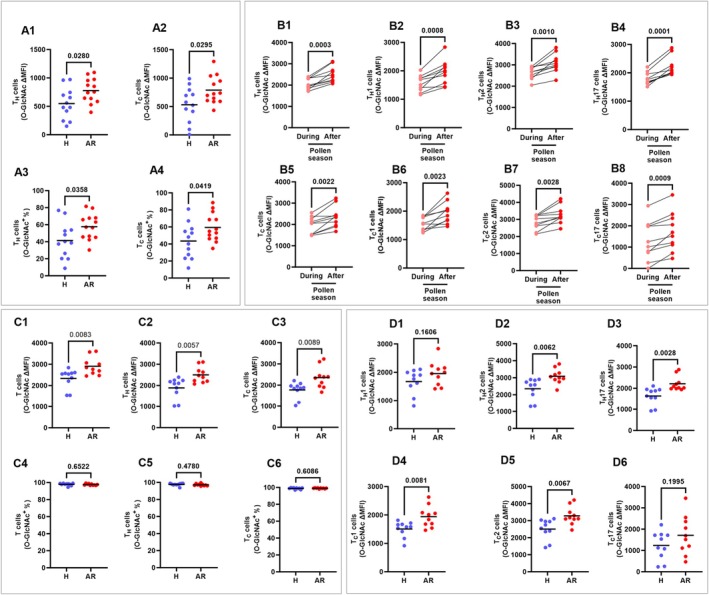
O‐GlcNAcylation in circulating T‐cell subsets in allergic rhinitis. A1‐A4: Cohort 1 post‐season samples: O‐GlcNAc ΔMFI and %O‐GlcNAc^+^ in TH and TC cells in healthy controls versus AR patients. B1–B8: Cohort 2 paired samples of AR patients: O‐GlcNAc ΔMFI in TH, TH1, TH2, TH17, TC, TC1, TC2 and TC17 cells during versus after the pollen season. C1–C6: Cohort 2 unpaired post‐season: O‐GlcNAc ΔMFI and %O‐GlcNAc^+^ in total T, TH and TC cells in AR versus controls. D1–D6: O‐GlcNAc ΔMFI in TH1, TH2, TH17, TC1, TC2 and TC17 subsets post‐season in AR versus controls. ΔMFI = MFI‐isotype control. Statistics: Paired or unpaired Student's *t*‐test.

Paired sampling of AR patients in Cohort 2 revealed a clear seasonal pattern. Across all eight examined T‐cell subsets including total TH, TH1, TH2, TH17, total TC, TC1, TC2 and TC17, O‐GlcNAc was consistently higher after the natural pollen season than during (Figure 1B). This within‐person longitudinal change indicates that O‐GlcNAc dynamically reflects natural allergen exposure and more strongly reflects post‐season immune activation than peak‐season responses. Importantly, the frequencies of these subsets remained stable across time points, confirming that the differences reflect intracellular modulation rather than shifts in cell composition.

To further assess off‐season immune alterations and strengthen the paired findings, we analysed unpaired post‐season samples from Cohort 2 using an extended permeabilisation and intracellular staining protocol. Total T, TH and TC cells again demonstrated significantly higher O‐GlcNAc ΔMFI in AR patients than in controls (Figure 1C1–C3). As expected from the extended protocol, intracellular staining efficiency was near‐complete, resulting in O‐GlcNAc^+^ frequencies approaching 100% across all groups (Figure 1C4–C6). Subset‐level analysis revealed increased O‐GlcNAc in TH2, TH17, TC1 and TC2 cells in AR patients, whereas TH1 and TC17 cells did not differ from controls (Figure 1D). Frequencies of all examined subsets were comparable between groups, confirming that these elevations represent true intracellular increases in O‐GlcNAc rather than population shifts.

Mechanistically, O‐GlcNAc integrates metabolic and immunological signals through the balance between O‐GlcNAc transferase (OGT) and O‐GlcNAcase (OGA), influencing T‐cell receptor signalling, cytokine production and transcriptional programmes [3, 5].

Elevated O‐GlcNAc in AR may reflect sustained T‐cell activation following allergen exposure, altered glucose metabolism, or prolonged maintenance of effector‐memory populations [6]. The dynamic seasonal modulation observed here suggests that O‐GlcNAc may serve as an objective cellular indicator of ongoing allergic inflammation [7, 8].

One possible explanation for the higher post‐season levels is that highly activated O‐GlcNAc‐high T cells are preferentially recruited to nasal mucosa during peak pollen exposure and subsequently recirculate into peripheral blood as inflammation subsides. By linking O‐GlcNAc to persistent post‐season T‐cell activation, this study reveals a promising immunometabolic candidate biomarker that may help guide clinical assessment and future therapeutic approaches in allergic rhinitis.

Limitations include the modest sample size and reliance on peripheral blood. Future studies should assess whether similar patterns are present in nasal mucosa and evaluate correlations between O‐GlcNAc and symptoms, disease severity, or response to allergen immunotherapy. In addition, symptom scores were not systematically recorded at sampling visits, which restricts direct correlation between O‐GlcNAc and clinical disease activity. Nevertheless, the consistent findings across two independent cohorts support the robustness of this immunometabolic signature.

In summary, our data identify T‐cell O‐GlcNAc as a reproducible immunometabolic feature of allergic rhinitis, capturing both persistent off‐season inflammation and dynamic seasonal immune activation. These findings position O‐GlcNAc as a potential cellular biomarker of allergic disease activity and warrant further investigation into its diagnostic utility and therapeutic relevance.

Future studies should determine whether O‐GlcNAc levels correlate with symptom severity, treatment response, or mucosal inflammation. Such studies may help establish O‐GlcNAc as a clinically useful biomarker for monitoring allergic disease activity and guiding therapeutic interventions.

## Author Contributions

Conceptualisation: L.O.C., E.I.C. Data curation: E.I.C., L.O.C., M.J. Formal analysis: E.I.C. Investigation: E.I.C., O.W. Methodology: L.O.C., E.I.C. Project administration: L.O.C., E.I.C., M.J. Recourses: E.I.C., M.J., E.N., A.K., M.P., M.E. Supervision: L.O.C. Validation: E.I.C., L.O.C. Writing – original draft: E.I.C., M.J. Writing‐review and editing: E.I.C., M.J., E.N., A.K., M.P., M.E., O.W., L.O.C. All authors have confirmed the manuscript.

## Funding

This study was supported by Konsul Berghs Stiftelse, CIMED (The Center for Innovative Medicine), Region Stockholm (ALF) and The Swedish Heart Lung Foundation.

## Ethics Statement

All patients provided written informed consent before inclusion and all procedures and handling of patient information were conducted in accordance with the Declaration of Helsinki and the ethical permits approved by the Swedish Ethical Review Authority (Diary No. 2016/823‐31/2, 2021‐00325, 2021‐06514‐02).

## Conflicts of Interest

The authors declare no conflicts of interest.

## Data Availability

Data are available from the corresponding author upon reasonable request.
